# Cryopreservation of Embryos and Oocytes in Human Assisted Reproduction

**DOI:** 10.1155/2014/307268

**Published:** 2014-03-23

**Authors:** János Konc, Katalin Kanyó, Rita Kriston, Bence Somoskői, Sándor Cseh

**Affiliations:** ^1^Infertility and IVF Center of Buda, Szent János Hospital, Budapest 1125, Hungary; ^2^Faculty of Veterinary Science, Szent István University, Budapest 1078, Hungary

## Abstract

Both sperm and embryo cryopreservation have become routine procedures in human assisted reproduction and oocyte cryopreservation is being introduced into clinical practice and is getting more and more widely used. Embryo cryopreservation has decreased the number of fresh embryo transfers and maximized the effectiveness of the IVF cycle. The data shows that women who had transfers of fresh and frozen embryos obtained 8% additional births by using their cryopreserved embryos. Oocyte cryopreservation offers more advantages compared to embryo freezing, such as fertility preservation in women at risk of losing fertility due to oncological treatment or chronic disease, egg donation, and postponing childbirth, and eliminates religious and/or other ethical, legal, and moral concerns of embryo freezing. In this review, the basic principles, methodology, and practical experiences as well as safety and other aspects concerning slow cooling and ultrarapid cooling (vitrification) of human embryos and oocytes are summarized.

## 1. Introduction

The first successful mouse embryo cryopreservation (CP) was reported independently from each other by two research groups in 1972 [1–3]. One year later, the birth of the first calf from frozen embryo was published [4]. The first human pregnancy from frozen embryo was achieved with the same procedure used successfully for CP of mouse and cow embryos; however, it was terminated by spontaneous abortion in the 2nd trimester [5]. Since then, both sperm and embryo CP have become routine procedures in human assisted reproduction (AR) and oocyte CP is being introduced into clinical practice and is getting more and more widely used.

Embryo CP has decreased the number of fresh embryo transfers and maximized the effectiveness of the IVF cycle. Similarly, embryo CP is a crucial tool in cases of cancelled embryo transfer (ET) due to ovarian hyperstimulation risk, endometrial bleeding, elevated serum progesterone levels on the day of triggering, or any other unplanned events. There is still a large debate on the best stage, protocol/procedure, and cryoprotective additives (CPA) to use. The average potential of a frozen stored embryo to become a living child lies in the order of 4%, and babies born from cryopreserved embryos do not represent more than 8−10% of the total number of babies born from AR [6]. However, it is unquestionable that successful CP of zygotes/embryos has greatly enhanced the clinical benefits and cumulative conception rates possible for couples following a single cycle of ovarian stimulation and IVF. Results expressed as the augmentation of the delivery rate per oocyte harvest vary greatly in the literature, between 2% and 24% [7]. The data shows that women who had transfers of fresh and frozen embryos obtained 8% additional births by using their cryopreserved embryos [8, 9].

**Figure 1:**
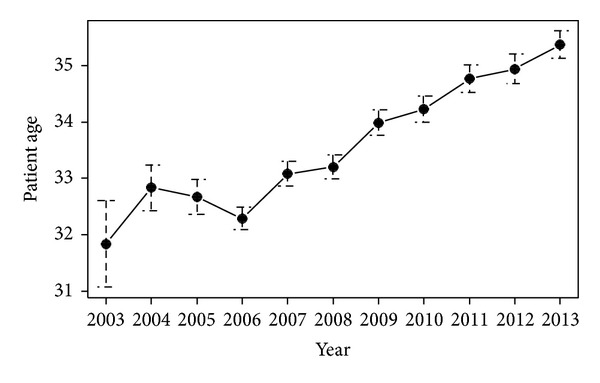
Increasing of average patient age in the last decade. Data are presented as mean ± SE.

The metaphase II (MII) oocyte has a very special structure (i.e., large size, very sensitive to low temperature, extremely fragile, high water content, low surface to volume ratio, presence of the spindle and other cell organelles, not optimal plasma membrane permeability to CPA and water, etc.) that leads to complex difficulties associated with its CP. The spindle is crucial for the events following fertilization in the completion of meiosis, second polar body formation, migration of the pronuclei, and formation of the first mitotic spindle. The damage (depolymerization) and/or absence of the spindle compromise the ability of the oocyte to fertilize and undergo normal preimplantation development. In addition, hardening of the zona pellucida—which is a consequence of CP—can adversely affect the normal fertilization process. However, oocyte CP offers more advantages compared to embryo freezing: (1) fertility preservation in women at risk of losing fertility due to oncological treatment, premature ovarian failure, or chronic disease; (2) it can help alleviate religious and/or other ethical, legal, and moral concerns of embryo storage; (3) it helps to overcome problems such as when the husband is unable to produce a viable sperm sample or when spermatozoa cannot be found in the testis at a given moment in case of nonobstructive azoospermia; (4) it makes “egg banks and/or egg donations” possible by eliminating donor-recipient synchronization problems; and (5) it allows women to postpone childbirth until a later time/age (e.g., after establishing a career, etc.). The latter is called social freezing when the oocytes are cryopreserved for nonmedical purposes. For about 10 years, in parallel with the technical improvement of oocyte freezing, the possibility of egg storing for nonmedical purposes is more extensively discussed and more commonly accepted by the general population and expert committees in the USA and Europe. The aim of the social freezing is to prevent age-related fertility decline which is widely promoted by fertility centers and the lay (unacademic) press throughout the world. It is a fact that the best reproductive performance/ability of women is around their 25–30 years of age. Afterwards pregnancy rates decline relatively fast from 35 years and miscarriage rates rise exponentially. After the age of 43 years, chances of becoming pregnant are very low [10, 11]. However, it is a worldwide tendency that women decide to give birth in their elder ages, as compared to earlier/20–30 years ago. Data of our patients having frozen cycle indicate that the average age (*n* = 3601) increased from 31.8 to 35.4 in the last 10 years (Figure 1).

**Figure 2:**
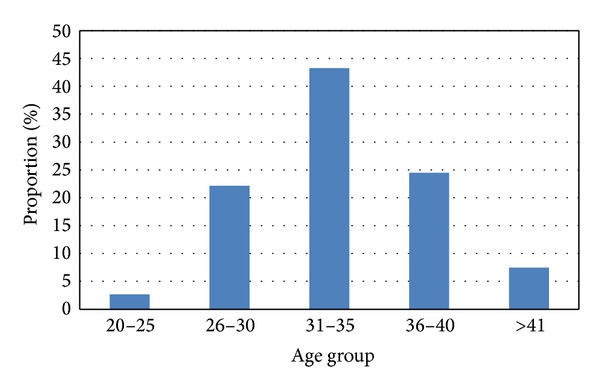
Proportion of age groups at our clinic.

In the case of almost 70% of the frozen cycles the patients were between 31 and 40 years old and 7.5% of them were >41 (Figure 2).

The “age effect” is detectable in the frozen embryo survival rate which slowly but continuously decreased in the last 10 years as the average age of the patients increased by 4 years without doing any modification in the freezing process (89% versus 81%; *P* < 0.0001). The number of successful frozen cycles is significantly lower over 30 years and there is a strong significant difference over 35 compared with under 30 years of age (*P* < 0.01 and *P* < 0.0002). The success rate of embryo/oocyte CP depends on several variables: efficacy of the freezing process, carriers used for vitrification (open versus closed), frequency of cycles with CP in the assisted reproductive program, the criteria for selection of embryos/oocytes for freezing, and the results of fresh embryo transfers. Results can be expressed as survival rates (but it is not enough alone, retention of normal physiological function of the cell organelles is essential), implantation rates, pregnancy rates, or delivery rates per transferred or thawed embryo s or harvested oocytes [12].

In this review, we summarize recent results including our own experiences concerning oocyte and embryo CP.

## 2. A Short Overview of the Basic Principles and Methodology of Slow Cooling and Vitrification

The traditional slow cooling methods for CP are referred to as* equilibrium cooling*, and the rapid/ultrarapid procedures (vitrification) as* nonequilibrium cooling* [13–15]. Various factors influence the survival of embryos and oocytes cryopreserved by equilibrium or nonequilibrium cooling procedures [8, 16].

## 3. Traditional Slow Cooling of Embryos and Oocytes

The greatest challenge during the CP of embryos and oocytes is to prevent the formation of ice crystal and toxic concentrations of solutes, which are the two main causes of cell death associated with CP, while maintaining the functionality of intracellular organelles and the viability of the embryo/oocyte. In order to do so the freezing solution, in which the cells are suspended, must be supplemented with cryoprotective additives (CPA). Exposure to CPA supports the dehydration of the cell and reduces intracellular ice formation. The CPA may be divided into two groups:* intracellular/membrane-permeating* (i.e., propylene glycol/PG/, DMSO, glycerol/G/, and ethylene glycol/EG/) and* extracellular/membrane-nonpermeating* compounds (i.e., sucrose, trehalose, glucose, amid, ficoll, proteins, and lipoproteins). The permeable CPA displaces water via an osmotic gradient and partly occupies the place of the intracellular water, while the extracellular CPA increases the extracellular osmolarity generating an osmotic gradient across the cell membrane supporting the dehydration of the cell before CP. At the same time, it prevents the rapid entry of water into the cell after thawing during rehydration/dilution out of the permeating CPA [8, 13–15].

Dehydration of the cell mainly depends on the permeability properties of the cell membrane. There are differences in permeability among the embryos of different species to water and permeating CPA. Embryos usually are less permeable to G than to PG or EG. Furthermore, the earlier the stage of development, the less permeable are the embryos [15–17]. The permeability properties of immature and mature oocytes differ and can vary by 7-fold between individual human MII oocytes [18, 19]. This difference in membrane permeability may have a strong impact on the outcome of slow freezing of oocytes but can be controlled by the elevation of the concentration of the nonpermeable CPA and the environmental temperature [20, 21]. By having the concentration of nonpermeating CPA increased (sucrose: 0.2 and 0.3 M) higher survival rates were reported, and the overall fertilization rates of frozen-thawed oocytes appeared to be similar to those of fresh oocytes [20, 22–28].

Prior to slow cooling, dehydration of the embryos/oocytes is carried out by exposure to a mixture of permeable and nonpermeable CPA (duration: 10 minutes). In the case of human embryos/oocytes, with very few exceptions, low concentration of PG (1.5 M) and sucrose (0.1–0.25–0.5 M) is used for early cleavage stage embryos and oocytes and G for blastocyst stage embryos. In case of the original successful CP protocol mouse and cow embryos were cooled with a slow cooling rate (between minus 0.3°C–0.5°C/min) to very low temperatures of minus 80°C–120°C [1–5]. Therefore, the duration of the procedure was very long (several hours). Willadsen [29] and Willadsen et al. [30] described a variation of this method in which sheep and bovine embryos were cooled slowly at a rate of 0.3°C/min, but only to minus 30–35°C before being plunged into liquid nitrogen (LN_2_) [29, 30]. With this modification the duration of the CP process was dramatically shortened (1.0–1.5 hours). Since then, this short protocol has become the treatment of choice for freezing of domestic animal embryos. Despite the excellent results achieved with animal embryos, human embryos are generally frozen with a low cooling rate of 0.3°C/min to about minus 30°C to 40°C, followed by an increased cooling rate of minus 50°C/min to a temperature of minus 80°C–150°C before being plunged into LN_2_ [7, 8]. During slow cooling, the dehydration process is thought to continue until minus 30°C, after which any remaining water is super cooled [14]. During the slow cooling phase ice nucleation (seeding) is induced manually between −5 and −8°C (close to the true freezing point of the solution).

Embryos/oocytes cooled slowly to subzero temperatures of minus 30°C to 40°C before being rapidly cooled to minus 196°C require rapid warming/thawing in warm water of 25°C–37°C [13, 17].

Rapid thawing is followed by removal of the CPA from the embryo/oocyte. Rehydration of the cells is carried out in decreasing concentrations of permeating CPA, generally in the presence of increased concentrations of nonpermeating CPA. A common practice is to dilute CPA out of the frozen embryo/oocyte in a stepwise fashion. The use of a nonpermeating solute, such as sucrose as an osmotic buffer, decreases the chances of an osmotic shock and shortens the duration of the process (see earlier) [8, 16, 27, 31, 32]. Long term storage of embryos and oocytes requires temperatures below minus 130°C, the glass transition temperature of water. In practice, the easiest and safest way is to store cryopreserved embryos/oocytes in LN_2_ at minus 196°C. Mouse model experiments indicate that the extended storage of embryos/oocytes does not affect the outcome of thawed cycles [17]. Live mice and sheep have been produced from cryopreserved embryos stored for more than 15 years in LN_2_ [17]. Children have been born from embryos that were cryopreserved for more than 8 and 12 years [33].

## 4. Vitrification (Ultrarapid Cryopreservation) of Embryos and Oocytes

Vitrification (i.e., a glass-like state) is an alternative approach to embryo/oocyte CP which has been recently described as a revolutionary technique; however, the first successful embryo vitrification was published in the middle of the 1980s [34]. Vitrification is different from slow freezing in that it avoids the formation of ice crystals in the intracellular and extracellular space [34]. Vitrification is the solidification of a solution by an extreme elevation in viscosity at low temperatures without ice crystal formation, a process achieved by a combination of a high concentration of CPA (4–8 mol/L) and an extremely high (ultrarapid) cooling rate [15, 35–37]. In contrast to slow freezing (when dehydration of the embryos/oocytes starts during the equilibration in the freezing solution prior to slow cooling and continues during slow cooling to minus 30–35°C), during vitrification, cells are dehydrated mainly before the start of the ultrarapid cooling by exposure to high concentrations of CPA, which is necessary to obtain a vitrified intracellular and extracellular state afterwards. The potential risk associated with the vitrification procedure is the high concentration of CPA that could be toxic to cells. However, it is possible to limit CPA toxicity by mixing different CPA, thereby decreasing the relative concentration of each CPA, and by reducing the exposure time of embryos/oocytes to the solution to a minimum [15, 34]. The freezing solutions that are commonly used for vitrification are composed of permeating (e.g., EG, G, DMSO, PG, acetamide; >4 M) and nonpermeating (e.g., sucrose, trehalose; >0.5 M) agents. In some protocols, the vitrification medium is also supplemented with macromolecules such as polyethylene glycol, ficoll, or polyvinylpyrrolidone [15, 34, 37]. By increasing viscosity, the macromolecules support vitrification with lower concentrations of CPA. In order to further increase the cooling rate (>10.000°C/min) necessary for successful vitrification, the volume of the solution in which the embryos/oocytes are vitrified has been recently dramatically decreased (0.1–2 *μ*L). To achieve this, special carrier systems (open versus closed) have been developed such as open pulled straws, Flexipet-denuding pipettes, Cryotop, electron microscopy copper grids, cryoloops, or the “Hemi-Straw” system [15, 35, 37, 38]. Closed systems have been developed for safety reasons. Comparing the open and closed systems Bonetti et al. [39] using closed carriers reported acceptable survival rates, but with multiple vesicles throughout the cytoplasm of oocytes which can be a likely consequence of not rapid enough temperature reduction in the closed system [39]. However, because of the improving results, the application of vitrification—especially for CP of human blastocyst and oocyte—has recently been greatly increased [15].

Technically vitrification is very difficult to perform, because of the very concentrated, viscous, and small volume of vitrification solutions in which the embryos/oocytes must be handled for only a very limited amount of time (<1 min) prior to and during vitrification. Therefore, in order to achieve the optimal/high survival rate the embryologist performing vitrification has to be very well trained. This is not the case in the case of slow freezing when the embryos/oocytes are cooled slowly (with a special cell freezer), because slow freezing is a more flexible technique. Similarly to slow freezing, rapid thawing is required for the optimal survival of vitrified embryos/oocytes, followed by stepwise rehydration using similar techniques employed after slow cooling.

## 5. Practical Experiences with Human Embryo Cryopreservation Using Slow Cooling and Vitrification

Generally, PG is used for the freezing of zygote and cleavage stage embryos and G for the CP of blastocysts [7, 8, 12, 35]. For many years, the preferred stages for human embryo CP were the zygote and early cleavage stages. Blastocyst freezing was abandoned for years, since only 25% of zygotes were able to reach the blastocyst stage in vitro in usual culture media, and overall low pregnancy rates were reported. Recently, new embryo culture systems—such as the coculture on feeder cells and the sequential media—have been developed making it possible to obtain good quality blastocysts in 50–60% of the cases [40]. Therefore, the importance of blastocyst CP increased in the last 8–10 years. Furthermore some of the published data indicate that human blastocysts obtained using sequential media appear to be only half as cryoresistant as the cocultured ones [7, 40–42].

Early cleavage stage embryos are considered surviving CP when they keep at least half of their initial blastomeres intact after thawing. The moderate loss of cells did not significantly influence implantation. In an early, large multicentre study with 14 000 cleavage stage slow frozen and thawed embryos it was determined that 73% of the embryos had at least half of their initial blastomeres still intact and the results showed clinical pregnancy and implantation rates of 16 and 8.4%, respectively, after transfer. In another study of over 300 single frozen embryo transfers of Day 2 embryos at the 4-cell stage and the embryos lost only a single blastomere during freezing/thawing (25%) similar implantation equivalent with fully intact frozen embryos and also with fresh embryos was obtained [25]. Data obtained from experience with slow cooling in 1.5 M PG plus 0.1 M sucrose is that around 75–85% of all cryopreserved cleavage stage embryos survive CP and that 50 to 60% of all thawed embryos will be totally preserved (100% of blastomeres survived). The lower survival rate of biopsied cleavage stage embryos could be improved by increasing the concentration of the nonpermeating CPA, sucrose prior to freezing [43]. Edgar et al. [44] observed that increasing the concentration of the sucrose from 0.1 M to 0.2 M resulted in a highly significant increase in survival [44]. Not only did the survival rate increase but the proportion of the fully intact embryos also significantly increased (54.6% versus 80.5%). The implantation rate per embryos thawed increased too, but it was not as significant (22.1% versus 17.5%). This modified slow freezing technology together with increased sucrose concentration has produced results which are equivalent to that of the best results obtained with vitrification.

The most widely used freezing solution for slow cooling of blastocysts is the combination of G and sucrose. The reported survival rates with a minimum of 50% survival of the inner cell mass and trophoblast cells are around 69%–98%, and the implantation rates are around 16%–30% [40–42]. Data indicates that the speed of development has influence to the survival rate. Reexpansion of frozen-thawed blastocysts in vitro is considered to be a very good sign of survival (70 to 80% of thawed blastocysts). A survival rate of 88% was reported for slow cooled blastocysts, whether or not they had been biopsied for PGD. In the same study the implantation rate was similar for fresh (34%) and thawed (35%) PGD blastocysts. Based on more than 400 frozen-thawed embryos Konc et al. [45] found no difference in the survival, implantation, and pregnancy rates of embryos cryopreserved on Days 3 and 5. However, in the pregnant group significantly higher implantation rate was observed with Day 5 blastocyst than with Day 3 embryos [45].

Early cleavage stage human embryos have been successfully vitrified in DMSO, EG, DMSO + sucrose, EG + sucrose and DMSO + EG + sucrose based solutions, and cca. 60%–80% of survival rate with at least 50% of their original blastomeres intact, and several pregnancies/deliveries have been reported (pregnancy rate: 10%–15%) [15, 46]. Kuwayama et al. [47] vitrified cleavage stage embryos with EG + DMSO + sucrose and the results showed a small but significant increase in survival (98% versus 91%), but no difference in the pregnancy rate relative to slow cooling was found [47]. In a similar comparative study no difference was found in the survival and implantation rates between slow cooling and vitrification [48]. Balaban et al. [49] using PG + EG + sucrose based solution observed higher survival (94.8% versus 88.7%) and a higher rate of fully intact embryos (73.9% versus 45.7%) in the vitrified group, compared with slow frozen Day 3 embryos which had been frozen in 1.5 M PG + 0.1 M sucrose [49]. The use of special carrier systems—through increased cooling speed—resulted in better survival and pregnancy rates after vitrification (survival rate of 90% and pregnancy rates of 25–60%). Kolibianakis et al. [50] in their review concluded that vitrification was not associated with a higher probability of pregnancy than slow freezing in experienced groups, but it did show a higher postthawing survival rate in cleavage and blastocyst stage embryos [50].

For blastocyst vitrification the most widely used solution is a mixture of EG and DMSO. Blastocysts have recently been successfully vitrified with improved survival rates in different carrier systems allowing ultrarapid cooling in small volumes of CPA solution. The reported overall survival rates are around 70–99% and the implantation rates are around 20–50% [51–55]. Ebner et al.   [56] having used closed system reported 74% survival and 39% implantation rates [56]. With another closed system the overall reported survival rate was 78%, with 56% of blastocysts fully intact after thawing. The implantation rate of the fully intact blastocysts was 16% compared to 6.4% in those with moderate damage [57]. Vanderzwalmen et al.  [58] published 86% survival rate and an implantation rate of 30% having used an aseptic vitrification system [58].

In a comparative study Kuwayama et al.  [47] found that the survival of vitrified blastocysts was slightly but significantly higher (90%) than that of slow cooled blastocysts (84%). However, pregnancy rates (53% versus 51%) and live birth rates (45 versus 41%) per transfer were not significantly different [47]. In a study with over 500 blastocysts in each group, Liebermann and Tucker [60] obtained no difference in the survival rate (96.5% versus 92.1%), in the pregnancy rate per transfer (46.1% versus 42.9%), and in the implantation rate (30.6% versus 28.9%) between vitrified and slow frozen groups [60].

## 6. Practical Experiences with Human Oocyte Cryopreservation Using Slow Freezing or Vitrification

Since the first successes achieved in the field of human oocyte CP many changes have been introduced into the slow cooling procedure. Increasing the sucrose concentration both in the slow freezing and vitrification solutions (from 0.1 M to 0.3 M) increased the rate of dehydration and the survival and fertilization rates of MII oocytes in a dose-dependent manner [20, 22–28]. Changing the temperature of the equilibration with CPA, ice nucleation (seeding) and plunging embryos into LN_2_, replacing sodium with choline (low sodium medium), or injecting sucrose directly into the cytoplasm of the oocyte all improved oocyte survival [32, 61, 62]. These results indicate that there is still room to improve the outcome of slow freezing of oocytes.

Slower development relative to fresh controls, both with respect to timing of the first cleavage division and the developmental stage reached on Day 2, has been observed in oocytes slowly cooled in 0.3 M sucrose [24, 63]. Konc et al. [22] reported comparable fertilization rates (fresh: 83%; frozen: 76%) but significantly slower development in the cryopreserved group, although implantation rates per embryo and oocyte were similar (fresh: 18% and 11%; frozen: 15% and 7%) [22]. Their results show a very pronounced difference in the cell stage on Day 2 between the frozen and fresh groups of oocytes (*P* < 0.05) as they found slower embryo development in the frozen oocyte cycles relative to fresh cycles. In the frozen group 64% of the embryos remained in the 2-cell stage and only 17% were in the 4-cell stage on Day 2. In contrast, in the fresh group on Day 2 66% of embryos were already in 4-cell stage and only 25% of them were in the 2-cell stage. Oocytes analyzed immediately after thawing displayed severe disorganization or disappearance of the spindle after slow freezing or vitrification. However, culturing oocytes for 1 to 3 hours after CP allows the spindle to repolymerize [11, 64–67]. Martinez-Burgos et al. [67] observed that vitrification seems to maintain the spindle apparatus at higher rates; therefore vitrified oocytes tend to repolymerize their spindles more effectively and faster than slow cooled oocytes; however, they showed higher misalignment between the meiotic spindle and the polar body [67]. Interestingly, they found no differences in DNA fragmentation between slow cooling and vitrification. Ciotti et al. [68] also reported that spindle recovery was faster in vitrified oocytes compared to slow cooled ones [68]. In contrast, Cobo et al. [64] found comparable spindle recovery from vitrification and slow freezing after 3 hours of incubation [64]. Konc et al. [70] investigated the spindle dynamics/displacement in frozen-thawed human oocytes. In each oocyte, prior to freezing and after thawing and 3 hours in vitro culture—just prior to ICSI—the presence and location of the spindle was determined with Polscope. Their results indicate that by observing the response of the individual oocytes the spindle does not always reform in its original position within the oocyte. After thawing and culturing the oocytes, they were able to visualize the spindle in 84.3% of the oocytes. However, they found that in half of the oocytes (53.1%) in which the spindle was rebuilt/visualized it was detected in a new location, not at the initial place, indicating that the spindle and the polar body move relative to each other [70].

The most widely used vitrification solution consists of a mixture of permeating (2.7 M EG and 2.1 M DMSO) and nonpermeating CPA (0.5 M sucrose). New data obtained with the improved vitrification techniques (i.e., decreased volume of vitrification medium and very rapid cooling speed) show an increase in the postthaw survival and fertilization rates of vitrified human oocytes which are comparable to the fresh control oocytes. Cobo et al. [71] published their findings from a randomized controlled trial of over 3000 fresh and 3000 vitrified oocytes (92.5% survival) in an oocyte donation program, confirming no detrimental effects of vitrification on subsequent fertilization, development, or implantation [71]. Others using the same vitrification protocol, also in oocyte donation programs, reported similar outcomes [72, 73]. Results obtained with the same technique in standard infertility programme showed a trend towards lower overall clinical outcomes from vitrified oocytes, especially over the age of 34 [74–76].

Comparing the results of slow freezing and vitrification we have to take into consideration that most of the published data generated by oocyte vitrification was obtained mainly by open systems and from oocyte donation programmes in which the egg donors were fertile and generally young women.

## 7. Safety and Other Aspects of Oocytes and Embryo Cryopreservation

The total number of children born worldwide after the fertilization of frozen and thawed oocytes is more than 1500 [77–79]. Studies indicate that pregnancies and infants conceived after oocyte CP do not present with increased risk of adverse obstetric outcomes or congenital anomalies [80]. No increase in the number of abnormal or stray chromosomes has been observed in the thawed oocytes [81]. In addition, no difference was found when comparing the incidence of chromosomal abnormalities in human embryos obtained from fresh and frozen oocytes [81, 82]. The follow-up study of 13 children born from frozen oocytes failed to reveal any abnormalities in karyotype or organ formation, mean age at delivery, and mean birth weight [83]. In another study no intellectual and/or developmental deficits were found in children conceived from cryopreserved oocytes [69, 83–85]. Despite the promising results, there are still concerns regarding the possibility of chromosomal aneuploidies or other karyotypic abnormalities, organ malformations or other developmental problems in offsprings; therefore, further follow-up studies with adequate numbers of patients involved are needed to clarify this very important question.

For patients, who are facing infertility due to chemotherapy/radiotherapy, oocyte CP is one of the few options available to keep their fertility potential [78, 86]. Thus, the standpoint of the Practice Committee of the Society for Assisted Reproductive Technology, the Practice Committee of the American Society for Reproductive Medicine, and the American Society of Clinical Oncology is that (1) oocyte CP holds promise for future female infertility preservation, (2) recent laboratory modifications have resulted in improved oocyte survival, fertilization, and pregnancy rates from cryopreserved oocytes, (3) no increase in chromosomal abnormalities, birth defects, or developmental deficits has been noted in the children born from frozen oocytes, and (4) oocyte CP should not be considered any more as an experimental technique and must be recommended to cancer patients only and carried out with appropriate informed consent.

At present, spermatozoa and embryos/oocytes are commonly frozen/stored in LN_2_ using straws/vials and newly developed open or closed carriers used for vitrification. Since the freezing container may leak or shatter during freezing, the potential for contamination of liquid nitrogen represents a real danger, especially in case of the “open carriers” developed for embryo/oocyte vitrification with ultrarapid cooling. The occurrence of cross-contamination during LN_2_ storage of biological material and subsequent cross-infection of patients has previously been demonstrated [87]. Viruses have previously been found to survive direct exposure to LN_2_, including vesicular stomatitis virus, herpes simplex virus, adenovirus, and papilloma virus [88]. There is also evidence of contamination of LN_2_ by other microorganisms, including a wide range of bacterial and fungal species [89]. Given the strength of the evidence of LN_2_ contamination by microbes and cross-infection in certain situations the possibility of contamination or cross-contamination during reproductive cell CP should be taken seriously. There are a number of relatively simple details and possible changes to CP procedures that can minimize the potential for contamination or cross-contamination of stored samples; for example, all patients and donors whose reproductive cells will be cryopreserved should be screened (e.g., HBV, HCV, HIV, etc.); it is highly recommended that the infected materials be stored in separate containers for each infection; instead of open systems, closed systems should be used for vitrification; finally, the storage container should be periodically emptied and cleaned [87, 90, 91]. However, in a comparative study all embryos cryopreserved in sealed straws and cryovials were free from viral contamination [87]. Transport of material vitrified in very small volumes may also raise questions related to its impact on survival [91].
